# Animal Disease Burden in Nigeria, 2006–2023

**DOI:** 10.1155/tbed/1694850

**Published:** 2025-12-22

**Authors:** Ahmad I. Al-Mustapha, Veronica Adetunji, Oluwaseun A. Ogundijo, Ismail A. Odetokun, Lateefah Oyafajo, Hauwa W. Abali, Muftau Oyewo, Ahmed Tijani Abubakar, Shuaibu Osu Muhammad, Deborah Adeolu Adetunji, Adesoji Odukoya, Anasin Haruna, Folashade Bamidele, Nusirat Elelu, Folorunso O. Fasina

**Affiliations:** ^1^ Department of Veterinary Services, Kwara State Ministry of Agriculture and Rural Development, Ilorin, Kwara State, Nigeria; ^2^ Department of Food Hygiene and Environmental Health, Faculty of Veterinary Medicine, University of Helsinki, Helsinki, Finland, helsinki.fi; ^3^ Department of Veterinary Public Health and Preventive Medicine, Faculty of Veterinary Medicine, University of Ibadan, Oyo, Nigeria, ui.edu.ng; ^4^ Department of Veterinary Public Health and Preventive Medicine, Faculty of Veterinary Medicine, University of Ilorin, Ilorin, Kwara State, Nigeria, unilorin.edu.ng; ^5^ Department of Food Science and Technology, Faculty of Basic and Applied Sciences, Osun State University, Osogbo, Osun State, Nigeria, uniosun.edu.ng; ^6^ Department of Veterinary Services, Yobe State Ministry of Agriculture and Rural Development, Damaturu, Yobe State, Nigeria; ^7^ Nigerian Field Epidemiology and Laboratory Training Program, Abuja, Nigeria; ^8^ Africa Centres for Disease Control and Prevention, Addis Ababa, Ethiopia; ^9^ Department of Veterinary and Pest Control Services, Federal Ministry of Agriculture and Rural Development, Abuja, FCT, Nigeria, fmard.gov.ng; ^10^ Department of Veterinary Public Health and Preventive Medicine, Faculty of Veterinary Medicine, Usmanu Danfodiyo University, Sokoto, Nigeria, udusok.edu.ng; ^11^ Food and Agriculture Organization of the United Nations (FAO), Rome, Italy, fao.org; ^12^ Department of Veterinary Tropical Disease, University of Pretoria, Pretoria, South Africa, up.ac.za

**Keywords:** animal disease burden, epidemic-prone diseases, highly pathogenic avian influenza, Nigeria, peste des petits ruminants, transboundary animal diseases, zoonoses

## Abstract

Decision‐makers in animal health require reliable, evidence‐based, timely, yet sensitive data to design disease contingency and preparedness plans, make informed decisions, and prioritize health interventions. Using Nigeria‐specific animal health disease reports from the World Animal Health Information System (WAHIS), a global animal disease reporting platform, between 2006 and 2023, we conducted descriptive statistics to summarize the animal disease burden. We used a zero‐inflated negative binomial (ZINB) regression model to forecast annual estimates of new outbreaks for the top 10 most reported diseases [Newcastle disease (NCD), infectious bursal disease (IBD), highly pathogenic avian influenza (HPAI), fowl typhoid, contagious bovine pleuropneumonia (CBPP), foot and mouth disease (FMD), African swine fever (ASF), peste des petits ruminants (PPR), rabies, and trypanosomiasis]. We analyzed 3362 official reports that were retrieved from the WAHIS platform and represented 9331 outbreaks of notifiable disease events that occurred in Nigeria. In summary, our analyses revealed that 3,248,945 cases and 4,911,495 culls were linked to the outbreaks, and ~2.2 million doses of vaccines were administered to animals. The most frequently reported non‐zoonotic diseases were IBD (446 outbreaks) in poultry, PPR (2836 outbreaks) in small ruminants, and 642 outbreaks of CBPP in cattle. During the period under review, 3822 outbreaks (10 diseases) were reported to be zoonotic. Based on the animal species, there were 10 poultry diseases with HPAI (1230 outbreaks), NCD (1605 outbreaks), and fowl typhoid (241 outbreaks) being most frequently reported. In cattle, there were 11 diseases, with CBPP (642 outbreaks) and trypanosomiasis (233 outbreaks) being the most prevalent. The data revealed spatial variations in disease burden, with 20.7% (1934 outbreaks) reported from North Central Nigeria. Using data from 2006 to 2023, our model forecasted an increasing annual trend in the number of NCD outbreaks from 413 (95% CI: 246–679) in 2025 to 772 (95% CI: 473–1283) by 2030. There was a moderate increase in forecasted estimates for the vaccine‐preventable diseases, rabies and FMD. The model forecasted some 68 FMD outbreaks (95% CI: 25–146) in 2025 and 144 (95% CI: 58–295) outbreaks by 2030 and Nigeria should plan against some 157 rabies outbreaks (95% CI: 79–289) in 2025, and this could rise to 252 outbreaks (95% CI: 133–457) by 2030. Outbreaks of ASF and the protozoan, tsetse‐fly‐transmitted disease trypanosomiasis were forecasted to have steady but slower increases, with ASF outbreaks estimated to range from 18 (95% CI: 5–46) in 2025 to 38 (95% CI: 13–93) by 2030y. Some 52 (95% CI: 19–112) outbreaks of the trypanosomiasis were forecasted for 2025. This is expected to increase to 95 (95% CI: 37–201) by 2030. The model estimated fewer than 10 (95% CI: 1–9) cases of HPAI annually. Finally, the model forecasted a modest but consistent rise in outbreaks of CBPP and fowl typhoid and a sharp increase in the outbreaks of PPR and IBD through 2030, mirroring gradual re‐expansion across the country. Our findings underscore the high animal disease burden in Nigeria despite potential underreporting, necessitating enhanced animal disease prevention and control strategies and increased investment in veterinary healthcare infrastructure. To ease the disease burden, Nigeria should implement syndromic surveillance, invest in regional diagnostic capacities, train community animal health workers, and establish an transdisciplinary One Health approach to disease surveillance.

## 1. Introduction

Epidemics occur when there is an increase, often sudden, in the occurrence of a disease in a specific geographical area [1]. The emergence of reportable and epidemic‐prone diseases (EPDs) in humans and animals may result in significantly higher morbidity, mortality, economic burdens (short‐term fiscal shocks and long‐term negative shocks in economic growth), psychosocial shocks, political instability, and other unforeseen consequences [2]. Such epidemics could be attributed to several factors, including changes in how humans live, increasing population density, expanded international travel and trade, rapid urbanization, and limited access to health care, as well as ecological changes, for example, incursion into wildlife areas and environmental degradation [2, 3].

In developing countries, the true burden of reported animal disease events and animal EPDs (AEPDs) is grossly underestimated due to varying local contextual factors such as weak surveillance, poor funding of animal health interventions, poor animal diagnostic facilities, and poor‐quality data [4]. These challenges limit the effective pre‐outbreak prediction, detection, response, and control of these diseases. In most instances, only zoonotic and a few trade‐sensitive transboundary animal diseases (TADs), which cause epidemics and pandemics, gain the attention of national policymakers, as well as international partners, to attract multinational funding [5–7]. For instance, after the 2023 anthrax outbreak (3 outbreaks and 48 cases), a multistate anthrax vaccination program was instituted [8].

In West Africa, open trans‐border movement of animals, goods, and humans, paucity of data on transhumance movement patterns, and the diversity of ecological zones pose threats to the region, particularly the Nigerian animal health sector [9]. Nigeria’s human population explosion and significant livestock population have resulted in increased incursion into previously uninhabited environments and more frequent human–livestock–wildlife contacts. These are associated with pathogen exposure for zoonotic diseases and epidemics in both humans and animals [10–12]. Furthermore, migratory birds, bats, and several other wildlife species have been shown to carry potential pathogens [13, 14]. Unlike the human surveillance system, Nigeria’s animal disease surveillance system (ADSS) is weak, neglected, and poorly funded [7].

There has been notable underreporting of animal diseases, and the magnitude of economic losses is substantial [15]. For instance, it was estimated that an outbreak involving 10% of the commercial bird population would cost Nigeria about $245 million [16]. Moreover, the mortality losses of the 2001 African swine fever (ASF) outbreak in 306 farms in Oyo State were estimated to be US$941,492 [17]. In 2013, the International Livestock Research Institute reported that the financial burden of five priority diseases (ASF, contagious bovine pleuropneumonia (CBPP), Newcastle disease (NCD), peste des petits ruminants (PPR), and trypanosomiasis) amounted to at least 29.2 billion Nigerian Naira (NGN) ($64 million) [18]. Furthermore, it is estimated that the cost of inaction against these diseases could be as high as NGN 10 billion for trypanosomiasis in cattle and pigs, NGN 8.9 billion for NCD in local chickens, NGN 6.9 billion for PPR in sheep and goats, NGN 2.2 billion for CBPP, and NGN 1.3 billion for ASF [18].

Nigeria is highly vulnerable to the effects of several AEPDs and ranks very high in the health burden of neglected zoonotic and tropical diseases [19]. There is therefore a need to provide evidence‐based inferences for future planning and improvement of the national animal disease surveillance and reporting system. Hence, the objective of our study was to evaluate the trends and patterns of reported animal diseases in Nigeria between 2006 and 2023, as a part of the proposed Risk Assessment for Animal Epidemic Prone Disease (RA‐4‐AEPD) project in the country.

## 2. Methods

### 2.1. Study Area

This study was conducted based on animal disease reports from Nigeria. The country is divided into six geopolitical zones: North Central (six states plus the Federal Capital Territory), North East (six states), North West (seven states), South East (five states), South South (six states), and South West (six states). The country’s livestock sector has experienced steady growth, with recent estimates indicating ~20.6 million cattle, 43.4 million sheep, 76.2 million goats, and 180.7 million chickens, making it one of the largest livestock hubs in West Africa [20]. Depending on the animal species, the majority of these livestock are managed under traditional extensive systems (especially ruminants), particularly in the northern regions, where pastoral and agro‐pastoral production remains dominant. The Fulanis, a widely dispersed ethnic tribe in West Africa known for pastoralism, manage ~90% of the country’s cattle population through seasonal transhumance, moving between a semi‐arid region, known as the Sahel, during wet seasons, and the Guinea savannah during dry seasons [21].

Cattle husbandry practices in Nigeria remain largely traditional, with limited adoption of modern technologies. More recently, partnerships in the milk value chain have increased cattle breeding programs to boost milk production in the country. The indigenous cattle breeds, particularly the White Fulani (Bunaji), Red Bororo (Rahaji), and Sokoto Gudali, are well‐adapted to the local environment but show relatively low productivity compared to exotic breeds. Poultry and swine husbandry are also crucial sectors of animal production in Nigeria. Unlike cattle, these animals are mostly raised intensively to reduce the turnaround time and increase profitability. However, challenges such as inadequate veterinary services, limited access to quality feed, and poor storage facilities continue to constrain the sector’s development, with annual meat production meeting only about 60% of the national demand [22].

### 2.2. Data Source

The data analyzed in this study were obtained from the open‐access portal of the World Animal Health Information System (WAHIS) at https://wahis.woah.org/#/dashboards/qd-dashboard. The WAHIS platform is an interactive global animal health reference database accessible via an internet‐based portal that processes data on animal diseases in real time and has three main elements: an early warning system, a monitoring system, and an information system for annual reporting. This study retrieved all the reports (bi‐annual) originating from Nigeria from its index report to the WAHIS platform from January 2006 until June 2023, covering any animal disease events within the 117 WOAH‐listed terrestrial animal diseases. All reports from Nigeria were included in this study, while reports not captured within the Nigerian geographical boundary were excluded.

One of the specificities of WAHIS is that all information must be confirmed by the competent sanitary authority of the notifying country before it is published. This, though well‐intentioned, is in itself a limitation, especially in resource‐limited countries where diagnostics are not readily available. In addition, data quality issues, systemic and technical barriers, and socioeconomic disparities among reporting countries/regions could be other limitations of data from the WAOH–WAHIS platform. Nevertheless, official information published in WAHIS is particularly useful for trade purposes, as evidence‐based for planning interventions, and to alert other international organizations to support countries affected.

### 2.3. Data Analysis

Nigeria submitted a total of 3364 validated animal disease events as reports to the WAHIS during the period under review. Following an initial screening, two entries in 2006 were excluded from the analysis because they were registered on the WAHIS platform as Nigeria, but no specific administrative region (state) was indicated. Hence, 3362 were processed and analyzed. For qualitative variables, the descriptive statistics were reported as frequencies and proportions, and for quantitative data, the mean was calculated. All analyses were conducted using the Statistical Package for Social Sciences (SPSS *v*.29.0.2.0, Armonk, NY, USA). Heat (spot) maps were created in QGIS (v. 3.30.2).

We estimated a 6‐year forecast (2025–2030) for each of these disease cases using Bayesian zero‐inflated negative binomial (ZINB) regression models, which account for both overdispersion and excess zeros (underreporting) common in our disease count data. Separate models were specified for each disease using Markov chain Monte Carlo (MCMC) sampling with four chains of 1000 iterations. The models incorporated the year as a continuous predictor, standardized to improve convergence. Parameter estimation was conducted using Hamiltonian Monte Carlo sampling with a target acceptance rate of 0.9. Model convergence was assessed using the R‐hat statistic, with all values below 1.1 indicating satisfactory convergence. Forecasts for 2025–2030 were generated from the posterior predictive distribution, with uncertainty quantified using 95% highest density intervals. All analyses were performed using PyMC v5.0 with ArviZ for diagnostic evaluation, ensuring robust probabilistic estimates that appropriately capture the inherent uncertainty in disease surveillance forecasting. Here, we present the forecast for the top 10 reported diseases (highly pathogenic avian influenza [HPAI], NCD, ASF, foot and mouth disease [FMD], rabies, and trypanosomiasis). We selected this model (as against others such as zero‐inflated Poisson, negative binomial, Tobit, or Poisson regression) because we believed that they were very flexible for real‐world outbreak data and better at handling variability between years and diseases.

## 3. Results

### 3.1. Animal Disease Reports

A total of 3362 official reports, comprising 9331 outbreaks of notifiable events that occurred in Nigeria between 2006 and 2023, were retrieved from the WOAH‐WAHIS platform on 30 June 2023. These outbreaks occurred in 1,049,884 animals of various species according to the considered diseases. Cumulatively, our descriptive statistics revealed that the number of cases was 3,248,945, which resulted in the culling and disposal of 4,911,495 carcasses of multiple species. The most important animal disease events reported are presented in Table 1. Based on the animal species involved, most official reports to the WOAH were attributable to 11 diseases of cattle, followed by 10 diseases of farmed poultry, which included 499 reports from commercial and domesticated poultry, including two reports of infection with HPAI viruses from the square‐tailed nightjar. The most frequently reported poultry diseases were HPAI, NCD, infectious bursal disease (IBD), and fowl typhoid. In small ruminants, PPR was the most frequently reported disease, whereas CBPP, trypanosomiasis, and FMD were the most frequently reported diseases in cattle (Table 1).

**Table 1:** Animal hosts of reported diseases in Nigeria, January 2006–June 2023.

Animal species	Non‐zoonotic			Zoonotic		
	Disease	Number of reports	Number of outbreaks	Disease	Number of reports	Number of outbreaks
Poultry (*n* = 10)	Gumboro (Infectious bursal disease)	207	446	Highly pathogenic avian influenza	499	1230
	Avian mycoplasmosis^a^	47	132	Newcastle disease	354	1605
	Pullorum disease	25	36	—	—	—
	Avian infectious bronchitis	16	30	—	—	—
	Marek’s disease	6	8	—	—	—
	Avian infectious laryngotracheitis	4	4	—	—	—
	Fowl cholera	4	2	—	—	—
	Fowl typhoid	136	241	—	—	—

Cattle (*n* = 11)	Contagious bovine pleuropneumonia	284	642	Trypanosomiasis^b^	111	233
	Lumpy skin disease	60	116	Bovine tuberculosis	46	99
	Foot and mouth disease	52	509	Brucellosis (*B. abortus*)	45	30
	Bovine babesiosis	26	59	Anthrax	2	3
	Hemorrhagic septicemia	12	8	—	—	—
	Bovine anaplasmosis	8	27	—	—	—
	Paratuberculosis	2	6	—	—	—
	Bovine viral diarrhea	1	1	—	—	—

Goats and Sheep (*n* = 5)	Peste des petits ruminants	680	2836	—	—	—
	Goat and sheep pox	38	137	—	—	—
	Contagious caprine pleuropneumonia	22	47	*Brucella melitensis*	2	4
	*Salmonella abortus ovis*	1	2	—	—	—

Swine (*n* = 2)	African swine fever	110	180	*Brucella suis*	2	2
Canines (*n* = 2)	—	—	—	Rabies	319	602
	—	—	—	*Echinococcus granulosus*	1	1
Equines (*n* = 2)	Equine influenza	14	14	—	—	—
	African horse sickness	4	4	—	—	—
Camelids (*n* = 1)	—	—	—	Camelpox	2	16
Leprae (*n* = 1)	Rabbit hemorrhagic disease	6	22	—	—	—
	Total	1765	5509	Total	1381	3822

^a^Avian mycoplasmosis *– Mycoplasma gallispeticum and M. synoviae*.

^b^ *Trypanosoma brucei*, *T. congolense*, *T. simiae*, *T. vivax*, *T. evansi*, and tsetse‐transmitted trypanosomiasis.

### 3.2. Zoonotic vs. Non‐Zoonotic Diseases

Some 1517 reports of 11 endemic zoonotic diseases were reported to the WOAH–WAHIS platform during the period under review. The reports were the result of 3822 outbreaks of these zoonotic diseases. Outbreaks of infection with the HPAI virus were the most widely reported, being reported in 36 states of the Federation, except for Cross River and Ondo States, and with the highest burden being in the states of Plateau (306 outbreaks), Kano (250 outbreaks), and Lagos (137 outbreaks). Rabies in dogs (319 reports and 602 outbreaks) and African animal trypanosomiasis (111 reports and 233 outbreaks) were the second and third most prevalent zoonotic diseases in Nigeria, respectively (Figure 1a). The endemic, mildly zoonotic poultry disease NCD (354 reports from 1605 outbreaks) and fowl typhoid (136 reports from 241 outbreaks) were the most frequently reported.

Figure 1(a) Map of Nigeria showing reported zoonotic diseases, 2006–2023. HPAI, Highly pathogenic avian influenza. (b) Map of Nigeria showing reported non‐zoonotic diseases, 2006–2023. (c) Animal disease events reported to the WAHIS platform from Nigeria, 2006–2023. AHS, African horse sickness; ASF, African swine fever; Avian IB, avian infectious bronchitis; Avian IL, avian infectious laryngotracheitis; Avian MY, avian mycoplasmosis; B. anaplasmosis, bovine anaplasmosis; B. babesiosis, bovine babesiosis; BVD, bovine viral diarrhea; CBPP, contagious bovine pleuropneumonia; FMD, foot and mouth disease; F. typhoid, fowl typhoid; HS, hemorrhagic septicemia; IBD, infectious bursal disease; LSD, lumpy skin disease; NCD, Newcastle disease; Para–TB, paratuberculosis; PPR, peste des petits ruminants; RHD, rabbit hemorrhagic disease; *S. abortusovis*, *Salmonella abortus ovis*.

**Figure:**
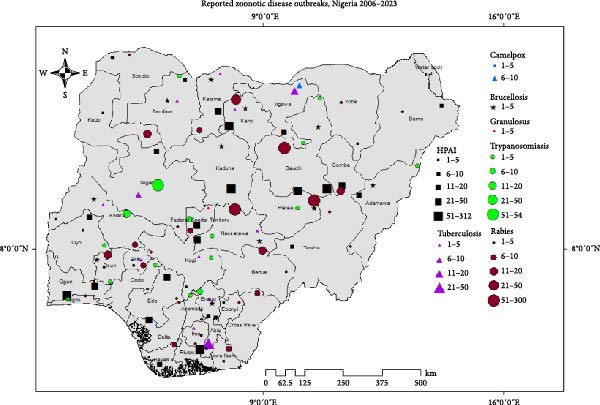
(a)

**Figure:**
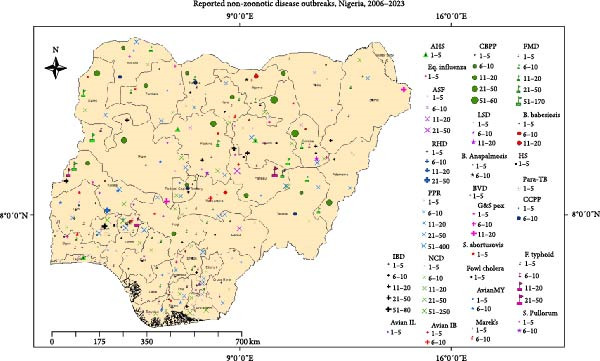
(b)

**Figure:**
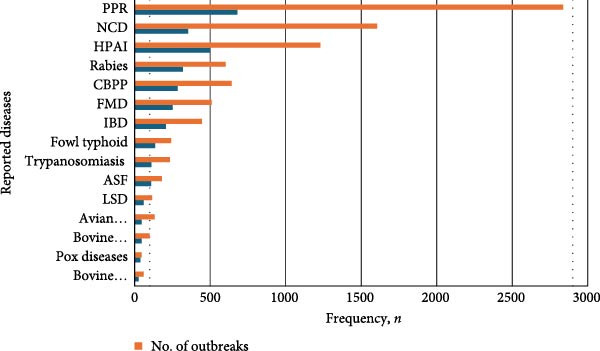
(c)

Cumulatively, 1765 reports of non‐zoonotic reportable terrestrial animal diseases were submitted in the period under review. These reports accounted for 5509 outbreaks of AEPD and other reportable disease. The most frequently reported non‐zoonotic diseases were PPR (680 reports from 2836 outbreaks), CBPP (284 reports from 642 outbreaks), and FMD (52 reports from 509 outbreaks) in cattle, as well as IBD (207 reports from 446 outbreaks) (Figure 1b,c).

### 3.3. Spatial Variations in Reportable Animal Diseases Across Nigeria

**Figure 2:**
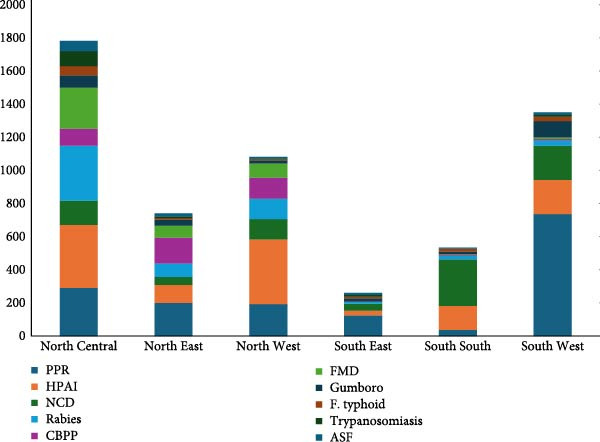
Variations in the top 10 animal disease outbreaks across the geographical regions of Nigeria.

Approximately 26.15% (*n* = 879) of all the reported animal diseases were from the North Central region (*n* = 1934 outbreaks), and 63.5% (*n* = 9331) of all outbreaks were from the three northern regions (North West, North Central, and North East), whereas the three southern regions (South West, South East, and South South) accounted for 36.5% of all outbreaks (*n* = 3406) (Figure 2). The highest detection and reporting of PPR was in the South West region of Nigeria (736 outbreaks), followed by 200 and 290 outbreaks from the North East and North Central regions, respectively. The states in northern Nigeria had the highest reporting of HPAI, with a total of 391 outbreaks reported from the North West region. In addition, 381 outbreaks (96 reports) and 206 outbreaks (70 reports) were reported from the North Central and Southwest regions, respectively. As an endemic poultry disease with severe economic implications, there were widespread reports of NCD from the six regions. More CBPP, FMD, and ASF cases were also reported from states in northern Nigeria (Figure 2).

### 3.4. Animal Disease Prevention and Control Strategies

Several disease prevention and control strategies were instituted to limit the spread of these reportable TADs across the country. These strategies include reactive vaccinations, in which a total of 2,186,299 doses of animal vaccines were administered to animals. Vaccinations against NCD (Lasota and Komarov) represented 89.1% of the vaccines administered. In addition, other poultry vaccines, such as the fowl typhoid and IBD vaccines, were also used to control the high economic consequences of these diseases in Nigeria’s poultry industry (Table 2). More than 123,000 doses of CBPP vaccine were administered to cattle, and ~60,000 doses of PPR were administered following positive confirmation of PPR across the country. HPAI was the only disease that was associated with the massive culling and disposal of ~5 million commercial poultry to prevent its further spread and possible spillover to humans.

**Table 2:** Reported disease prevention and containment strategies, January 2006–June 2023.

Diseases	Number of vaccinations	Number of animals killed and disposed	Number of animals slaughtered
African horse sickness	33	^a^	^a^
African swine fever	^b^	187	851
Brucellosis	2455	^a^	7
Contagious bovine pleuropneumonia	123,370	26	2991
Foot and mouth disease	^a^	5	292
Fowl typhoid	18610	125	459
Infectious bursal disease	29162	256	756
Highly pathogenic avian influenza	1500^c^	4,907,332	1748
Newcastle disease	1,948,218	2920	2184
Peste des petits ruminants	57,985	217	2896
Rabies	4963	338	25
Trypanosomiasis	^b^	192	77

^a^Unknown.

^b^No vaccine available.

^c^Nigeria has no vaccination policy against highly pathogenic avian influenza.

### 3.5. Temporal Variations in Animal Disease Reports

An average of 198 disease reports were submitted to the WAHIS–WOAH disease platform annually between 2006 and 2023. The lower range was 17 reports (from 17 outbreaks) between January and June 2023, and the upper range was 811 reports (2016 outbreaks) in 2021 (Figure 3a,b). Other years with high volumes of reports included 2020, with 306 reports (436 outbreaks), and 2008, with 291 reports (403 outbreaks). The finite analysis of the reports for the year 2021 revealed that most of the reports were due to PPR (408 outbreaks), NCD (477 outbreaks), HPAI (257 outbreaks), CBPP (133 outbreaks), FMD (154 outbreaks), rabies (147 outbreaks), and IBD (82 outbreaks), amongst others.

Figure 3(a) Reported outbreaks of NCD, IBD, HPAI, fowl typhoid, and ASF in Nigeria, 2006–2023, and a 6‐year forecast of their outbreak (2025–2030). ASF, African swine fever; HPAI, Highly pathogenic avian influenza; IBD, infectious bursal disease; NCD, Newcastle disease; (b) Reported outbreaks of FMD, CBPP, trypanosomosis, PPR, and rabies in Nigeria, 2006–2023, and a 6‐year forecast of their outbreak (2025–2030). CBPP, contagious bovine pleuropneumonia; FMD, foot and mouth disease; PPR, peste des petits ruminants.

**Figure:**
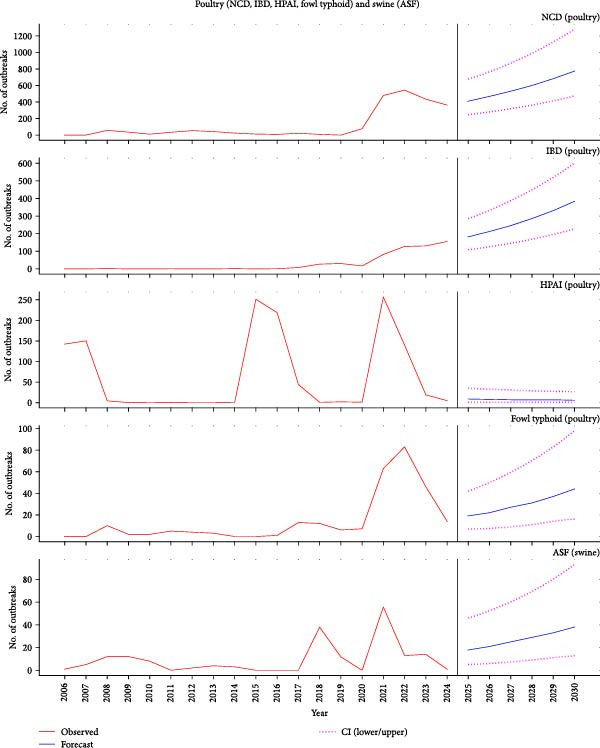
(a)

**Figure:**
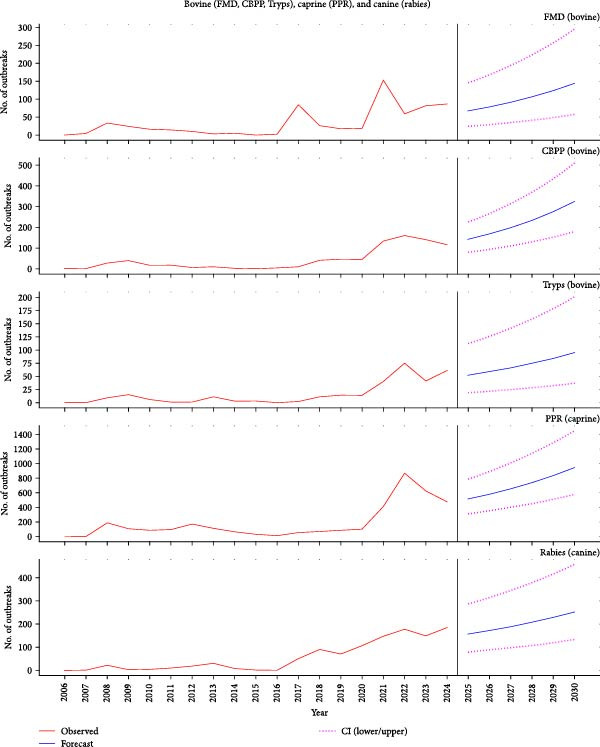
(b)

### 3.6. Disease Outbreak Forecasts

Using data from 2006 to 2024, our model forecasted an increasing trend in most of these diseases except for HPAI. For poultry diseases, the model forecasted that there would be some 413 (95% CI: 246–679) to 772 (95% CI: 473–1283) cases of NCD and less than 10 (95% CI: 1–9) cases of HPAI annually between 2025 and 2030, respectively. Some 18 (95% CI: 5–46) to 38 (95% CI: 13–93) outbreaks of ASF were forecasted annually. Among cows, some 52 (95% CI: 19–112) outbreaks of the protozoan, tsetse‐fly‐transmitted disease trypanosomiasis were forecasted for 2025. This is expected to increase to 95 (95% CI: 37–201) by 2030 (Figure 3a,b, Table 3). For the vaccine‐preventable disease, FMD, the model projected some 68 outbreaks (95% CI: 25–146) in 2025 and 144 (95% CI: 58–295) outbreaks by 2030. Finally, Nigeria should expect some 157 rabies outbreaks (95% CI: 79–289) in 2025, and this could rise to 252 outbreaks (95% CI: 133–457) by 2030. The model estimated fewer than 10 (95% CI: 1–9) cases of HPAI annually. The model forecasted a modest but consistent rise in outbreaks of CBPP and fowl typhoid and a sharp increase in the outbreaks of PPR and IBD through 2030, mirroring gradual re‐expansion across the country (Table 3). The model estimated some 143 CBPP outbreaks (95% CI: 79–226) in 2025, which could rise to 324 outbreaks (95% CI: 179–510) in 2030. Similarly, the forecast revealed that some 19 (95% CI: 7–42) to 44 (95% CI: 16–98) outbreaks of fowl typhoid would occur in Nigeria between 2025 and 2030.

**Table 3:** Projections of new outbreaks of the top 10 reportable animal diseases in Nigeria, 2025–2030.

Disease		2025	2026	2027	2028	2029	2030
Newcastle disease	Forecast	413	468	530	601	681	772
	UCL	679	765	867	986	1123	1283
	LCL	246	279	318	362	414	473
Highly pathogenic avian influenza	Forecast	9	8	8	7	7	6
	UCL	35	32	30	29	27	26
	LCL	1	1	1	1	1	1
African swine fever	Forecast	18	21	25	29	33	38
	UCL	46	52	60	69	80	93
	LCL	5	6	7	9	11	13
Foot and mouth disease	Forecast	68	79	92	107	124	144
	UCL	146	168	193	223	257	295
	LCL	25	30	35	42	49	58
Trypanosomiasis	Forecast	52	59	66	75	84	95
	UCL	112	126	141	158	178	201
	LCL	19	22	25	28	32	37
Rabies	Forecast	157	172	190	208	229	252
	UCL	289	316	345	378	416	457
	LCL	79	88	97	108	120	133
CBPP (contagious bovine pleuropneumonia)	Forecast	143	168	198	233	275	324
	UCL	226	266	313	368	433	510
	LCL	79	93	110	129	152	179
PPR (peste des petits ruminants)	Forecast	512	579	653	738	834	942
	UCL	789	891	1007	1137	1284	1450
	LCL	313	353	399	451	509	575
Fowl typhoid	Forecast	19	22	27	31	37	44
	UCL	42	50	59	70	83	98
	LCL	7	8	10	11	14	16
IBD (infectious bursal disease)	Forecast	182	212	245	285	330	383
	UCL	286	331	385	446	518	601
	LCL	108	125	145	168	195	226

Abbreviations: LCL, lower critical (predictive) limits; UCL, upper critical (predictive) limits.

## 4. Discussion

This study profiled the outbreaks of zoonotic and TADs in Nigeria and could be invaluable in assisting contingency planning and in making informed decisions in public and animal health. This work is important, as livestock production supports the livelihoods of over 600 million smallholder farmers and 30 million pastoralists in Africa [23, 24]. The occurrence of reportable EPDs in animals poses a severe public health threat (zoonotic transmission to humans) with economic consequences [25].

The weak disease surveillance and poor animal health diagnostics, among other issues, make Nigeria’s current disease reporting underperform, with similar trends in other low‐ and middle‐income countries in the world. The reported animal disease metrics, such as the number of animals vaccinated and the number of animals culled do not reflect the true animal disease burden in Nigeria. For instance, anecdotal report suggested that 70,000 pigs died or were culled/slaughtered due to ASF in Nigeria in 2020 [26], but this was not reported to the WAHIS platform. The results also reveal that 2,184,799 doses of vaccines were administered over 18 years. We believe the over‐the‐counter sale of veterinary biologicals, including vaccines, in Nigeria allows livestock owners to self‐vaccinate, again resulting in gross under‐reporting of vaccination parameters. The bottlenecks in Nigeria’s ADSS produce inaccurate estimates of the disease burden at the community, state, and national levels, which limits the ability to control these diseases. This passive surveillance on farms and in communities is also hindered by a poor diagnostic capacity [27]. Therefore, investment in regional diagnostic laboratories, point‐of‐care diagnostics, and the adoption of a One Health approach that fosters collaboration between human, animal, and environmental sectors could make animal disease surveillance in resource‐limited settings more accessible, cost‐effective, sustainable, and connected across geographical scales [28, 29]. Another important factor that likely contributes to the proliferation and spread of TADs in Nigeria is the fear of income and livelihood loss, resulting from the reporting of animal health issues, as compensation and state support initiatives are limited.

In addition, more TADs than zoonoses were reported during the period under review. Spatially, there was an uneven distribution regarding the evaluated disease reports, with the North Central, South West, and North West regions reporting the most outbreaks. Our study affirmed the association between the spatial patterns of these diseases with the distribution of vulnerable animal populations and/or the available diagnostic laboratories. Previous evaluations have confirmed spatial clustering of veterinary diagnostic services around veterinary facilities, and in our evaluation, most reports came from the states of Plateau, Kano, and Lagos, with the main veterinary diagnostic facilities Nigerian Veterinary Research Institute (NVRI) or their zonal offices [30].

Our data revealed that HPAI, rabies, and trypanosomiasis were the most frequently reported zoonotic diseases. This is in agreement with the findings of Ihekweazu et al. [7], who reported that these diseases ranked 1st, 2nd, and 7th in the list of prioritized zoonotic diseases in Nigeria. Timely and effective integrated surveillance and control programs for these and other underreported zoonoses and TADs would counter both the human and animal disease threats. Such surveillance programs should target the environment, food products, wildlife, humans, and domestic animals using a comprehensive One Health approach [31]. Generally, the zoonotic diseases reported in this study pose significant economic, food safety, and public health challenges to Nigeria’s livestock and GDP. Infection with the HPAI viruses H5N1 and H5N8 represents the most common of the zoonotic avian influenza viruses (AIVs) in Nigeria [32]. While H5N1 has been the most commonly reported AIV subtype included in the WAHIS reports, other subtypes of emerging strains, such as H5N8 transmitted by migratory wild birds into the country, have also been reported across the country. In addition, Laleye et al. [33] reported the first detection on the African continent of the H5Nx clades 2.3 and 4.4 b, which are related to European viruses, in samples collected during active HPAI surveillance and reported outbreaks in Nigeria.

Rabies was the second most frequently reported zoonotic disease, with outbreaks reported in 31 out of the 37 states (FCT included). It is a VPD for which a national rabies control strategy was launched in 2022. There is an ongoing national mass vaccination campaign against rabies across all states of Nigeria, in line with the global target to eliminate human rabies by 2030. To achieve the “Zero by 30” targets for rabies, there is a need to adhere to the set stepwise approach and periodically review the goals, and adequate financing must be provided to ensure the attainment of at least 70% herd immunity against rabies in dogs and cats, to ensure that human‐mediated rabies is stemmed in Nigeria [34, 35].

Although no vaccinations have been developed for human or animal trypanosomiasis, preventive measures against the disease center around the control of the tsetse fly, the main vector for trypanosomes. The occurrence of more than 100 reports of trypanosomiasis underscores the need for a control program. Furthermore, there was gross underreporting of brucellosis, with only 36 reported outbreaks. A recent meta‐analysis of human and animal brucellosis reported a national seroprevalence of 17.6% (554/3144) and 13.3% (8547/64,435), respectively [36]. It should be noted that diseases such as brucellosis can be subclinical and do not cause massive deaths. Hence, pastoralists and animal health practitioners may underprioritize its reporting through the official animal disease reporting system. Brucellosis infection in animals is associated with massive economic losses due to a decreased calving rate, delayed calving, culling due to infertility, treatment costs, decreased milk production, abortions, stillbirths, the birth of weak calves, and the loss of man‐hours in infected people [37]. Although a VPD, vaccinations against brucellosis remain uncommon in Nigeria, mainly due to the poor risk perception and underdiagnosis of the disease [38].

Of the non‐zoonotic diseases reported to the WAHIS platform from Nigeria, PPR was the most common and accounted for more than 2836 outbreaks in small ruminants. In cattle, significant disease containment efforts are needed to curb outbreaks of CBPP, FMD, and lumpy skin disease, which are associated with significant losses of production in affected herds. IBD also represents a significant threat to Nigeria’s poultry industry with outbreaks in poultry across the country [39]. In pigs, outbreaks of ASF cause significant losses to farmers and are associated with significant loss of GDP in Nigeria [17, 32, 40]. All of these diseases are vaccine‐preventable, except for ASF (Supporting Information: Table S1). Currently, Nigeria has national control programs against PPR and CBPP (and has started piloting FMD), especially with the support of the WOAH vaccine bank and the increased vaccine production capacity of the NVRI (Vom). Most of the other reported diseases are also vaccine‐preventable, making vaccination an effective means to reduce their burden and associated economic costs. For some VPDs, such as rabbit hemorrhagic disease (RHD), the federal government has no policy, so it is at the discretion of the farmer to vaccinate or not based on the local epidemiology of the disease [41]. These VPDs cause severe economic losses to farmers and affect the livelihood of rural women who keep these animals as a means of sustenance [42, 43]. Among pastoralist settlements (*n* = 336) in seven states of northern Nigeria, Bolajoko et al. [44] reported that animal disease events caused significant losses via mortalities, with high overall mortality rates of both cattle (15.3%) and small ruminants (30.9%) from various diseases. In addition, the study recorded reproductive losses of 8.7% and 16.6% in cattle and small ruminants, respectively.

These TADs are not unique to Nigeria, as most of them have also been reported in other countries in Sub‐Saharan Africa [45]. For instance, the new H5N8 HPAI subtype was traced to Southern Africa [33], whereas an outbreak of RHD was linked to rabbits imported from the Benin Republic, a neighboring country [41, 46]. However, no RHD outbreaks in the Benin Republic have been reported to the WAHIS platform since 2016. In addition, a study from Ghana revealed that over 6000 rabbits were lost as a result of an RHD outbreak in 2019 [47], but there were no official reports to the WAHIS platform. These examples highlight the need for improved surveillance, regional collaboration, and an effective reporting system to safeguard the lives and livelihoods of livestock owners.

The wide variability in disease outbreaks across Nigeria could be attributed to several socioeconomic factors, as more animal‐specific outbreaks were reported in specific areas. For instance, Lagos and Kano are the hubs for HPAI in Nigeria (these states also have a high population of poultry and poultry markets), whereas more ASF and PPR cases were reported in pig and small ruminant hubs in the central and southern parts of the country, respectively. The increasing number of reports over the years under review could be attributed to the efforts of federal epidemiologists and disease surveillance agents employed under the REDISSE project [48]. The development of control strategies (policy documents) against priority TADs in 2022 is a step in the right direction. These policy documents, when fully implemented, could provide a legal framework and targets that, when achieved, could help protect Nigeria’s livestock sector [49, 50].

We believe that the forecasted estimates of outbreaks for the 10 diseases [NCD, HPAI, IBD, and fowl typhoid (poultry), CBPP, FMD, and trypanosomiasis (cattle), rabies (dogs), PPR (small ruminants), and ASF (pigs)] should enhance Nigeria’s disease preparedness plans. Policymakers could utilize this information to prioritize preventive practices, such as reactive vaccinations against these diseases, while enhancing general farm biosecurity measures and notifying animal health workers to improve their active case search in the field. The disease forecasts must, however, be interpreted with caution, as several socio‐demographic factors could have influenced the forecasts. There are very few studies that have forecasted animal disease burden in Nigeria due to the wide variability and uncertainty involved. Despite this, our forecast agreed with the finding of a study that reported an increasing trend in PPR cases in Nigeria by 2030 [51].

Despite the gross under‐reporting, a 2016 estimate for the 10‐year global burden of epidemics put the cost at over US$600 billion or ~0.7% of global income. The impact of these AEPDs is not only limited to the health impact associated with the immediate outbreak but extends to more inherent and enormous, often underestimated economic consequences [2]. Such economic impacts often affect low‐income families, who keep animals for sustenance and whose livelihoods depend on them [52, 53].

The ADSS in Nigeria, like in many other Sub‐Saharan African countries, experiences challenges with funding and sustainability, thus contributing to issues with detection of outbreak events, data quality, and response [25, 27, 54]. While no surveillance system is perfect (detects all cases of an event), the ADSS in Sub‐Saharan Africa must be strengthened to improve its sensitivity, specificity, coverage, cost–benefit relationship, timeliness, positive predictive value, accuracy, simplicity, flexibility, completeness, and acceptability [25]. In addition, several sociodemographic challenges often influence the ability of the ADSS to act as an early warning system to detect disease events. These challenges result in a long time to detection (the number of days between the initial introduction of a notifiable disease onto a farm and the CVO of the nation learning of its presence) [55].

This study is subject to certain limitations, and the analyzed data may not be comprehensive enough. In addition, we may have missed many cases that were not reported through the official veterinary authorities due to the following: (1) little or no surveillance in the wildlife sector, (2) the inadequate quantity and quality of the trained workforce in the livestock sector nationally and regionally in most low‐ and middle‐income countries, and (3) user‐friendly electronic data capture tools may not have been utilized consistently over the years (2006–2023). In addition, there is a lack of interoperability between the different reporting platforms that have been used in the subnational system and the data transmitted to the national system [56, 57].

## 5. Conclusion

This study analyzed Nigeria’s animal disease data and revealed 9331 outbreaks of 10 zoonotic and 23 non‐zoonotic reportable animal diseases from 2006 to 2023, shedding light on the significant burden these diseases pose to public health and the economy. We forecasted annual estimates of outbreaks for the top 10 diseases to enable policymakers to prioritize preventive measures. Our findings underscore the high animal disease burden in Nigeria, necessitating enhanced animal disease prevention and control strategies and increased investment in veterinary healthcare infrastructure. To ease the disease burden, it is essential to implement syndromic surveillance, invest in training community animal health workers, establish an transdisciplinary One Health approach to disease surveillance (at the sub‐national level), and improve regional diagnostic capacities (including point‐of‐care diagnostics).

## Ethics Statement

This study does not require ethical approval.

## Consent

The authors have nothing to report.

## Conflicts of Interest

The authors declare no conflicts of interest.

## Author Contributions

**Ahmad I. Al-Mustapha**: conceptualization, data curation, formal analysis, writing–draft, writing–review.**Veronica Adetunji**: conceptualization, writing–draft, writing–review. **Ismail A. Odetokun**: conceptualization, formal analysis, writing–draft, writing–review. **Lateefah Oyafajo**: formal analysis and creation of spot maps, writing–review. **Oluwaseun A. Ogundijo**: conceptualization, writing–draft, writing–review. **Hauwa W. Abali**: data curation, writing–draft, writing–review. **Muftau Oyewo:** data curation, writing–draft, writing–review. **Deborah Adeolu Adetunji**: data curation, writing–draft, writing–review. **Ahmed Tijani Abubakar**: conceptualization, data curation, writing–draft, writing–review. **Adesoji Odukoya**: data curation, writing–draft, writing–review. **Anasin Haruna**: data curation, writing–draft, writing–review. **Folashade Bamidele**: data curation, writing–draft, writing–review. **Nusirat Elelu**: conceptualization, writing–draft, writing–review. **Folorunso O. Fasina**: conceptualization, data curation, formal analysis, writing–draft, writing–review. **Veronica Adetunji**: writing–draft, writing–review.

## Funding

Open access publishing facilitated by Helsingin yliopisto, as part of the Wiley ‐ FinELib agreement.

## Supporting Information

Additional supporting information can be found online in the Supporting Information section.

## Supporting information


**Supporting Information** Table S1: Summary of vaccine‐preventable diseases in animals reported from Nigeria, 2006–2023.

## Data Availability

The disease reports are openly available at WAHIS.
