# Supplementary material for: Animal Disease Burden in Nigeria, 2006–2023
Source: Transbound Emerg Dis. 2025 Dec 22;2025:1694850. doi: 10.1155/tbed/1694850 (PMC12721471; doi:10.1155/tbed/1694850)
Supplement: Supplementary file 1 — Supporting Information Table S1: Summary of vaccine‐preventable diseases in animals reported from Nigeria, 2006–2023. [file TBED-2025-1694850-s001.docx]

**Animal Disease Burden in Nigeria, 2006–2023**

Table S1. Summary of vaccine-preventable diseases in animals reported from Nigeria, 2006–2023.

| **Disease** | **Animal hosts** | **Availability of vaccine** | **Vaccines readily available in Nigeria** | **Control programme in Nigeria** |
| --- | --- | --- | --- | --- |
| **Non-zoonotic diseases** | | | | |
| Infectious bursal disease | Avian | Yes | Yes | No |
| Avian mycoplasmosis | Avian | Yes | No | No |
| Pullorum disease | Avian | Yes | Yes | No |
| Avian infectious bronchitis | Avian | Yes | Yes | No |
| Marek's disease | Avian | Yes | Yes | Mostly at hatcheries |
| Avian infectious laryngotracheitis | Avian | Yes | No | No |
| Fowl cholera | Avian | Yes | Yes | No |
| CBPP | Cattle | Yes | Yes | Yes (National) |
| LSD | Cattle | Yes | Yes | No |
| FMD | Cattle | Yes | Yes | Yes (Sub-national) |
| Haemorrhagic septicaemia | Cattle | Yes | Yes | No |
| Paratuberculosis | Cattle | Yes | No | No |
| Bovine viral diarrhoea | Cattle | Yes | No | No |
| Peste des petits ruminants | Caprine | Yes | Yes | Yes |
| CCPP | Caprine | Yes | No | No |
| *Salmonella abortus ovis* | Ovine | Yes | No | No |
| African swine fever | Swine | No | No | Yes |
| African horse sickness | Equine | Yes | No | No |
| Rabbit haemorrhagic disease | Leprine | Yes | Yes | No |
| **Zoonotic Diseases** | | | | |
| HPAI | Avian | Yes | No | No vaccination policy |
| Zoonotic tuberculosis | Cattle | Yes | No | No |
| Newcastle disease | Avian | Yes | Yes | Yes (Sub-national) |
| Fowl typhoid | Avian | Yes | Yes | No |
| Brucellosis (*B. abortus and B. melitensis*) | Cattle | Yes | Yes | No |
| Rabies | Canine | Yes | Yes | Yes (National) |
| *Echinococcus granulosus* | Canine | Yes | No | No |
| Goat and Sheep pox | Caprine | Yes | No | No |
| Camelpox | Camelid | Yes | No | No |

HPAI – Highly pathogenic avian influenza, CBPP – Contagious bovine pleuropneumonia, LSD – Lumpy skin disease, FMD – Foot and mouth disease, CCPP – Contagious caprine pleuropneumonia. Sub-national control – Refers to vaccination activities organized locally either by the government or NGOs within a community, local government area or state.
